# Icariin-mediated structural stabilization of TNF-α triggers a pro-regenerative signaling switch for endothelial recovery after myocardial infarction

**DOI:** 10.3389/fphar.2026.1817340

**Published:** 2026-05-08

**Authors:** Ce Cao, Zixin Liu, Lili Yang, Jianshu Song, Lei Li, Jianhua Fu, Jianxun Liu

**Affiliations:** 1 Institute of Basic Medical Sciences of Xiyuan Hospital, China Academy of Chinese Medical Sciences, Beijing Key Laboratory of Chinese Materia Pharmacology, National Clinical Research Center of Traditional Chinese Medicine for Cardiovascular Diseases, Beijing, China; 2 Duke-NUS Medical School, Singapore, Singapore

**Keywords:** endothelial cell, heart failure, icariin, regeneration, TNF

## Abstract

**Background:**

Coronary microvascular recovery is essential for cardiac repair post-myocardial infarction (MI), but the endogenous signals driving endothelial cell (EC) cycle reentry remain elusive. While tumor necrosis factor-alpha (TNF-α) is typically considered a pro-inflammatory mediator, its biological effects are profoundly influenced by protein conformation. In this study, we identify a novel pro-regenerative signaling switch where structural stabilization of TNF-α, rather than its inhibition, dictates endothelial neogenesis.

**Methods:**

A rat model of HF was established via left anterior descending coronary artery ligation. *In vitro*, coronary microvascular endothelial cells (CMECs) were subjected to oxygen-glucose deprivation and reoxygenation. Bioinformatics analysis, molecular docking (MD) and molecular dynamics simulations (MDs) were employed to identify potential targets. The effects of Icariin (ICA) on EC proliferation, migration, and the TNF signaling pathway were validated using EdU assays, wound healing assays, and Western blotting.

**Results:**

Bioinformatics analysis and experimental validation identified the TNF signaling pathway as a critical regulator of EC regeneration. ICA significantly improved cardiac function and promoted CD31^+^/BrdU^+^ endothelial cell proliferation in the peri-infarction area of HF rats. MD and MDs revealed that ICA directly binds to the hydrophobic pocket of TNF-α, enhancing its structural stability. Mechanistically, ICA treatment upregulated the expression of cell cycle-promoting proteins by stabilizing TNF signaling, whereas TNF inhibition significantly abrogated the pro-proliferative effects of ICA. Our findings suggest that ICA may promote endothelial regeneration and alleviate HF in part by structurally stabilizing TNF-α and activating the downstream cell cycle cascade.

**Conclusion:**

This study provides a mechanistic basis for the ethnopharmacological use of the traditional herb *Epimedium* by demonstrating that its primary bioactive monomer, ICA, acts as a potential candidate for revascularization therapy.

## Graphical Abstract

**Figure:**
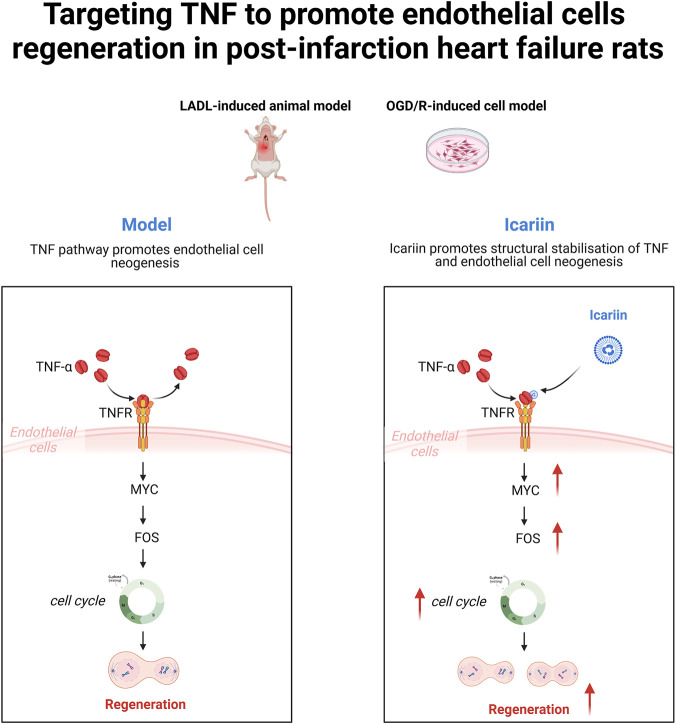
Created in BioRender. Xin G. (2026) https://BioRender.com/io9acr1.

## Highlights

- TNF-α structural stabilization drives coronary microvascular neogenesis.
- Icariin acts as a molecular stabilizer of the TNF-α trimer.
- The TNF-α-c-MYC-CDK4 axis is essential for endothelial cell cycle reentry.
- Targeted TNF knockdown exacerbates cardiac injury and impairs regeneration.
- Conformational modulation of cytokines offers a new strategy for cardiac repair.

## 1 Introduction

Heart failure (HF) following myocardial infarction (MI) is a significant global health concern, driven by complex pathophysiological processes that severely impact cardiac function and patient prognosis. The impairment of endothelial cells (EC) proliferation is a key factor contributing to the progression of HF after MI, as it affects the repair and regeneration of damaged cardiac tissues (Lu et al., 2025). Insufficient angiogenesis directly compromises the survival of residual cardiomyocytes and the efficacy of tissue repair (Narimani et al., 2025). Besides, proper endothelial-mediated vascularization can modulate the recruitment and activation of fibroblasts, thereby influencing the extent of pathological fibrosis (Ytrehus et al., 2018). Understanding the mechanisms underlying EC proliferation and identifying potential therapeutic targets are crucial for developing effective treatment strategies.

The tumor necrosis factor (TNF) signaling pathway has been implicated in various cardiovascular diseases (Huang et al., 2022; Ma et al., 2024). TNF, a pro-inflammatory cytokine, plays a dual-edged role in the heart. In the early stages of MI, the immune system is activated, and TNF is released as part of the innate immune response (Chen et al., 2022; Nash et al., 2019). It acts as a powerful mediator, triggering a cascade of events that are initially beneficial for the damaged heart. For instance, TNF can upregulate the expression of adhesion molecules on EC, promoting the recruitment of immune cells such as neutrophils and macrophages to the site of injury (Xie et al., 2024). These immune cells are essential for clearing damaged tissue debris and initiating the repair process. Moreover, TNF is involved in the regulation of the cell cycle in ECs. It can stimulate the proliferation of endothelial progenitor cells, which are crucial for the formation of new blood vessels during the process of angiogenesis (Hu et al., 2024). By enhancing the availability of these progenitor cells, TNF may help restore blood flow to the infarcted myocardium, thereby facilitating tissue repair (Kotyla, 2018; Sinagra et al., 2013; Papamichail et al., 2023). However, when TNF signaling becomes excessive or dysregulated, it can lead to a series of detrimental effects. Prolonged exposure to high levels of TNF can induce apoptosis in myocardial cells by activating a complex network of intracellular signaling pathways that ultimately trigger the activation of caspases, key enzymes responsible for programmed cell death (Müller-Ehmsen and Schwinger, 2004). This myocardial cell apoptosis further weakens the heart muscle, contributing to cardiac remodeling. In addition, overactive TNF signaling can exacerbate the inflammatory response in the heart (Sarzi-Puttini et al., 2005). It promotes the production of other pro-inflammatory cytokines, such as interleukin-1 (IL-1) and interleukin-6 (IL-6), thereby creating a vicious cycle of inflammation (van Loo and Bertrand, 2023; Levin et al., 2016). This chronic inflammation can damage the extracellular matrix (ECM) in the heart, disrupt the normal structure and function of the myocardium, and further impede endothelial proliferation (Han et al., 2024; Bradley, 2008; Horiuchi et al., 2010). However, the specific contribution of TNF signaling to EC proliferation during HF has not been fully clarified.

Traditional Chinese medicine (TCM) has a long-standing history in treating various diseases, and its potential in cardiovascular disease management has attracted increasing attention (Zhang et al., 2021; Xu et al., 2019; Wu et al., 2019; Chan and Ng, 2020; Huang et al., 2021; Li et al., 2021). TCM is composed of a vast number of natural compounds, which may offer novel therapeutic opportunities through their unique mechanisms of action. Among them, screening for specific compounds that can modulate the TNF signaling pathway to promote EC proliferation represents a promising research direction.

In this study, we aimed to comprehensively investigate the role of the TNF signaling pathway in EC proliferation in the context of HF after MI. Through a series of *in vitro* and *in vivo* experiments, we first sought to confirm the facilitatory effect of the TNF signaling pathway on EC proliferation. Using gene expression data from the Gene Expression Omnibus (GEO) database, we conducted bioinformatics analyses to identify key genes and pathways associated with this process. Our *in vitro* cell experiments, involving oxygen - glucose deprivation and reoxygenation models, provided direct evidence of the impact of TNF inhibition on EC survival and proliferation. *In vivo* animal experiments further validated these findings in a more physiological context, examining parameters such as infarct size, cardiac function, and biomarker levels. Furthermore, we employed molecular docking and simulation techniques based on the HERB database, which is a high-throughput experiment- and reference-guided database of traditional herbs used in Chinese medicine, to screen TCM-derived compounds that target TNF. This approach allowed us to identify potential candidates with high binding affinity to TNF. Among them, icariin (ICA) was selected for in-depth study due to its promising interaction with TNF. It is important to note that while *Epimedium* is the botanical herb traditionally utilized in clinical settings for cardiovascular ailments, ICA is its major pharmacologically active flavonoid. By focusing our molecular and cellular investigations specifically on the ICA monomer, we aimed to transition from broad ethnopharmacological observations to a precisely defined molecular mechanism. We investigated the effect of ICA on the structural stability of TNF through molecular dynamics simulations and explored its role in promoting EC proliferation via the TNF signaling pathway in *in vitro* cell experiments. The findings could contribute to the development of new treatment strategies to improve the prognosis of patients suffering from this debilitating condition.

## 2 Results

### 2.1 Number of endothelial cells regeneration is increased in post-infarction heart failure rats

**FIGURE 1:**
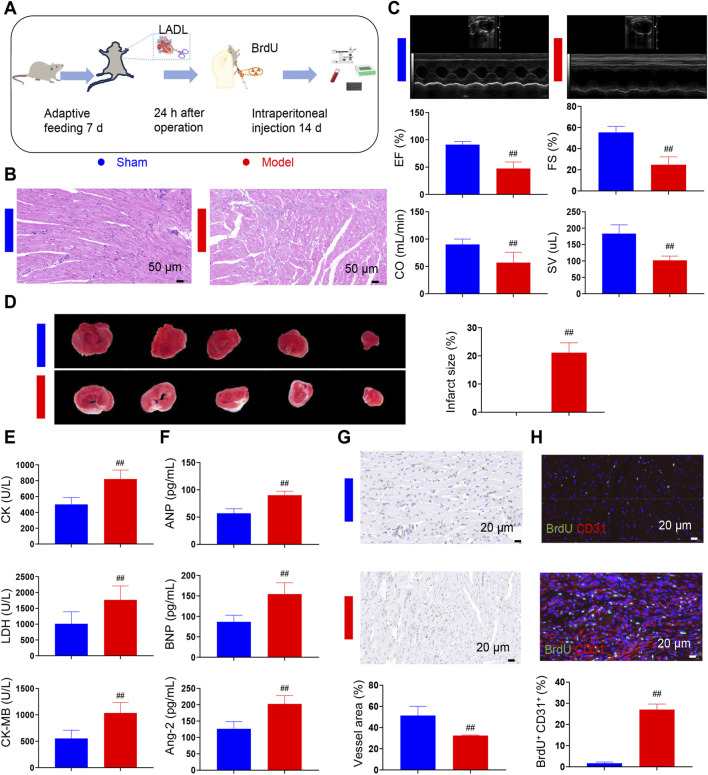
Increased number of endothelial cells proliferation in post-infarction heart failure rats. **(A)** Schematic diagram of *in vivo* animal experiments. **(B)** Pathological changes in each group (*n* = 3). **(C)** Echocardiographic changes in each group (*n* = 12). **(D)** Changes in infarct size in each group in rats (*n* = 6). **(E)** Determination of CK, CK-MB and LDH in rat serum (*n* = 12). **(F)** Determination of ANP, BNP and Ang-2 in rat serum (*n* = 12). **(G)** Immunohistochemical changes in post-infarction heart failure rats (*n* = 3). **(H)** Immunofluorescence assay for detection of regenerating endothelial cell (*n* = 3). LADL, left anterior descending coronary artery ligation. BrdU marks regeneration cells and CD31 marks endothelial cells. Data are presented as the means ± SD. Compared with sham group, ^#^ *p* < 0.05, ^##^ *p* < 0.01.

To comprehensively investigate the pathophysiological mechanisms underlying HF following MI in rats, a series of in-depth experiments were carried out. This experimental protocol successively encompasses a 7-day adaptive feeding stage, during which the rats’ physiological conditions are stabilized to ensure the reliability of subsequent operations (Figure 1A).

In the sham group, the myocardial tissue was observed to maintain a well-preserved and orderly architecture, with cardiomyocytes uniformly aligned and free of structural damage or pathological alterations. In contrast, in the model group, marked histopathological disruptions were evident, characterized by disorganization and fragmentation of myocardial fibers, along with a significant expansion of the interstitial space (Figure 1B). Echocardiographic analysis further confirmed these histological findings. As shown in Figure 1C, significant impairments in cardiac function were detected in the model group, as evidenced by marked reductions in ejection fraction (EF), fractional shortening (FS), cardiac output (CO), and stroke volume (SV) compared with the sham group. Moreover, as illustrated in Figure 1D, the infarct size was significantly increased in the model group, which further corroborated the extent of myocardial damage and its detrimental impact on cardiac performance. The levels of creatine kinase (CK), creatine kinase-MB (CK-MB), lactate dehydrogenase (LDH), atrial natriuretic peptide (ANP), brain natriuretic peptide (BNP), and angiopoietin-2 (Ang-2) were found to be significantly elevated in the model group compared with the sham group (Figures 1E,F). Beyond these biochemical parameters, vascular and cellular alterations were also examined. The vascular density in the model group exhibited marked changes, as illustrated in Figure 1G. Co-staining with bromodeoxyuridine (BrdU), a marker of proliferating cells, and cluster of differentiation 31 (CD31), a marker of ECs, revealed a significant increase in the number of regenerating ECs within the model group (Figure 1H). Notably, the increase in coronary microvascular endothelial cells (CMECs) was identified as a key compensatory mechanism in this pathological state.

### 2.2 TNF plays an important role in endothelial cell proliferation

**FIGURE 2:**
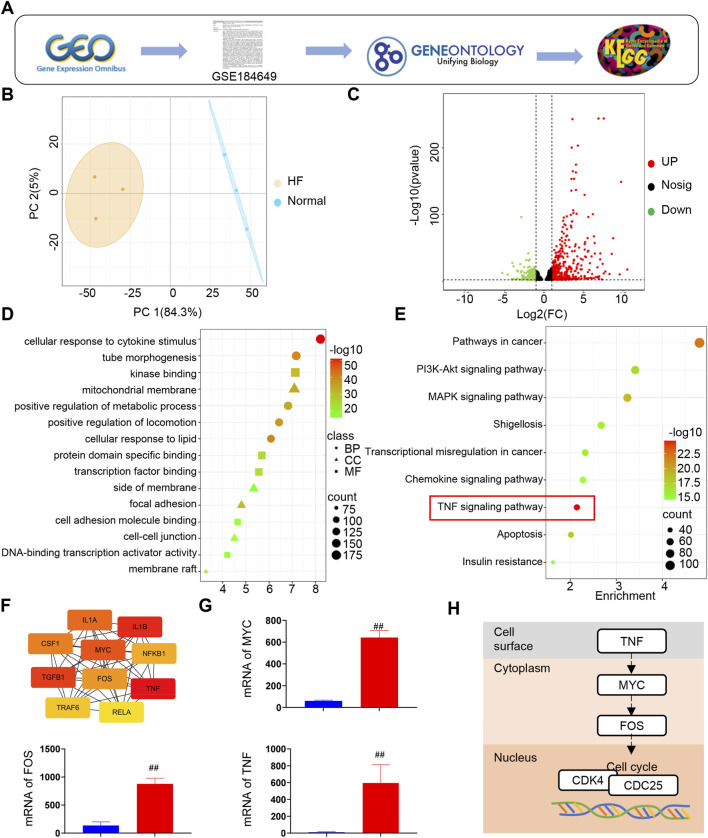
The TNF pathway plays an important role in heart failure. **(A)** Flowchart of the GEO database for online data retrieval. **(B)** Principal component analysis between groups. **(C)** Expression of differential genes between groups. **(D)** GO enrichment analysis of differential genes. **(E)** KEGG enrichment analysis of differential genes. **(F)** Enrichment of core genes of TNF signaling pathway. **(G)** mRNA expression of TNF, MYC and FOS. **(H)** TNF signaling pathway can regulate the cell cycle (*n* = 8).

To elucidate the specific mechanisms underlying EC proliferation in rats with HF following MI, a series of systematic bioinformatic analyses were performed using transcriptomic data retrieved from the GEO database (Figure 2A). Principal component analysis (PCA) clearly separated the HF group from the normal group, indicating distinct transcriptional signatures between the two conditions (Figures 2B,C). Gene Ontology (GO) analysis identified multiple relevant biological processes, molecular functions, and cellular components (Figure 2D), whereas Kyoto Encyclopedia of Genes and Genomes (KEGG) pathway enrichment highlighted several signaling pathways, among which the TNF signaling pathway was highly enriched, underscoring its potential importance in HF progression (Figure 2E). Notably, core genes such as IL1A, IL1B, CSF1, and MYC were enriched within the TNF pathway (Figure 2F), suggesting their potential interaction and contribution to pathway dysregulation in HF. Furthermore, KEGG mapping demonstrated that the TNF pathway regulates cell-cycle progression, wherein TNF activation at the cell surface initiates a signaling cascade involving MYC and FOS, ultimately influencing nuclear cell-cycle regulators such as CDK4 and CDC25 (Figures 2G,H). These findings collectively suggest that TNF-mediated modulation of the cell cycle represents a critical mechanism by which EC regeneration is regulated in HF following MI.

### 2.3 TNF signaling is indispensable for CMEC viability and proliferative capacity *in vitro*

**FIGURE 3:**
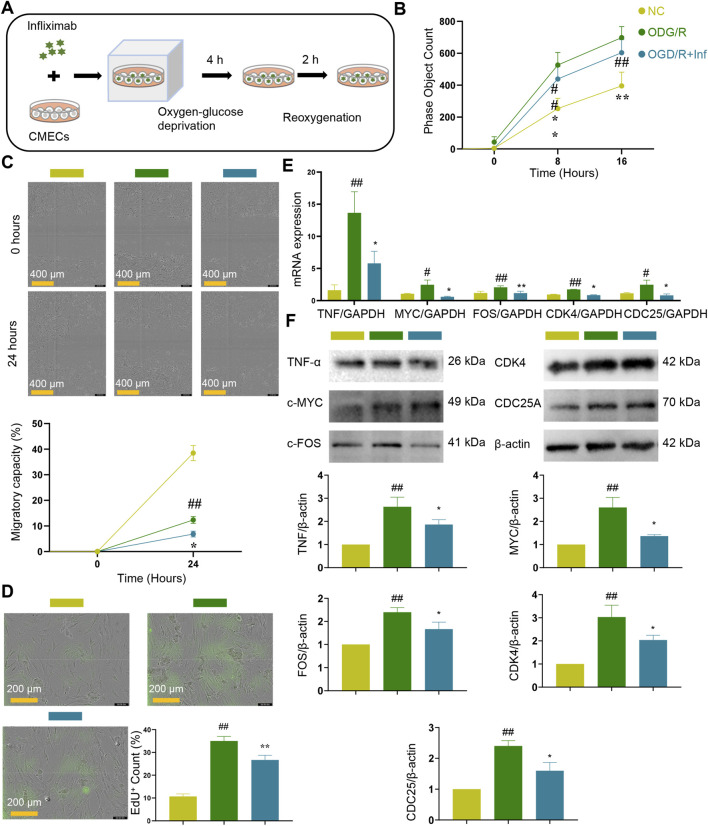
*In vitro* experiments confirm TNF signaling pathway promotes endothelial cell proliferation. **(A)** Schematic diagram of *in vitro* cell experiments. **(B)** Cell survival was reduced with the addition of TNF inhibitors (*n* = 6). **(C)** Migratory capacity was reduced with the addition of TNF inhibitors (*n* = 6). **(D)** EdU + cell was reduced with the addition of TNF inhibitors (*n* = 3). **(E)** TNF pathway mRNA expression was reduced by the addition of TNF inhibitors (*n* = 3). **(F)** Protein expression of TNF pathway was reduced by the addition of TNF inhibitors (*n* = 3). Data are presented as the means ± SD. Compared with normal control group, ^#^ *p* < 0.05, ^##^ *p* < 0.01. Compared with OGD/R group, ^*^ *p* < 0.05, ^**^ *p* < 0.01.

To validate the role of the TNF signaling pathway in promoting EC proliferation, a schematic of the *in vitro* experimental design was prepared (Figure 3A). CMECs were treated with infliximab, a TNF inhibitor, and subsequently subjected to oxygen–glucose deprivation/reoxygenation (OGD/R). Cell viability analyses demonstrated that TNF inhibition resulted in a significant reduction in CMEC survival (Figure 3B). Consistently, wound-healing assays revealed impaired migratory and reparative capacity in CMECs exposed to TNF inhibition (Figure 3C). In line with these findings, the number of EdU^+^ proliferating cells was markedly decreased following TNF pathway blockade (Figure 3D). Moreover, the mRNA expression levels of TNF pathway components were significantly downregulated upon infliximab treatment (Figure 3E), which was further corroborated at the protein level. The protein expression of TNF-α, c-MYC, c-FOS, CDK4, and CDC25A was significantly downregulated upon TNF inhibition (Figure 3F). Collectively, these data strongly indicate that TNF signaling is essential for CMEC survival, proliferation, and regeneration, and that its inhibition impairs endothelial proliferation.

### 2.4 TNF signaling is essential for CMEC survival and drives microvascular neogenesis *in vivo*

**FIGURE 4:**
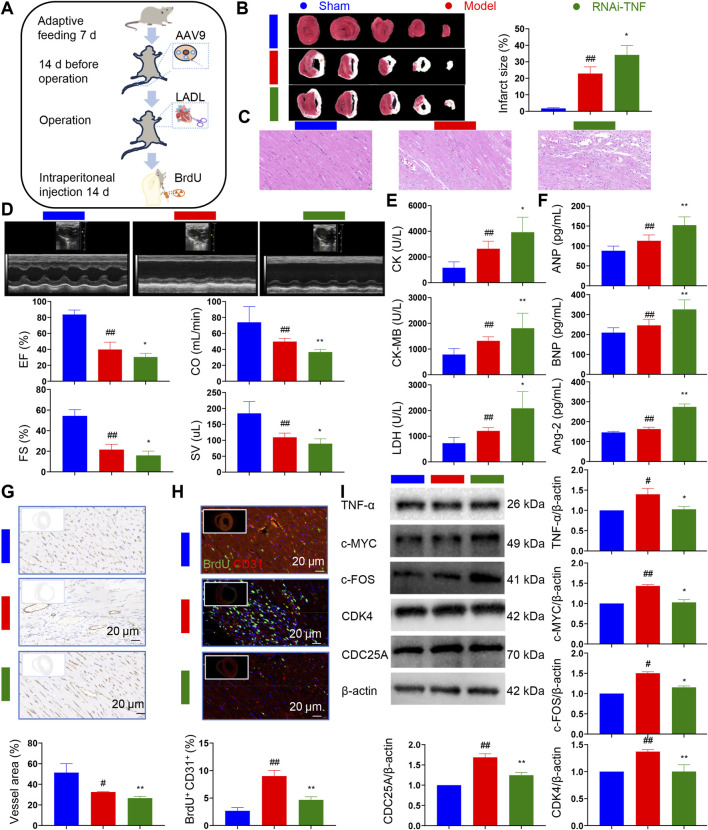
*In vivo* experiments confirm TNF signaling pathway promotes endothelial cell proliferation. **(A)** Schematic diagram of animal experiments. **(B)** Changes in infarct size in each group (*n* = 3). **(C)** Pathological changes in each group. **(D)** Echocardiographic changes in each group (*n* = 8). **(E)** Determination of CK, CK-MB and LDH in rat serum (*n* = 8). **(F)** Determination of ANP, BNP and Ang-2 in rat serum *(n* = 8). **(G)** Immunohistochemical changes in post-infarction heart failure rats (*n* = 3). **(H)** Immunofluorescence assay for detection of regenerating endothelial cell (*n* = 3). BrdU marks regeneration cells and CD31 marks endothelial cells. **(I)** Western blot of TNF signaling pathway in each group (*n* = 3). LADL, left anterior descending coronary artery ligation. Data are presented as the means ± SD. Compared with sham group, ^#^ *p* < 0.05, ^##^ *p* < 0.01. Compared with model group, ^*^ *p* < 0.05, ^**^ *p* < 0.01.

To further elucidate the role of TNF in angiogenesis *in vivo*, adeno-associated virus - 9 (AAV9) constructs targeting TNF were generated (Supplementary Figure S1; Supplementary Table S1). After establishing the HF model, RNAi-TNF was injected via the tail vein (Figure 4A). As expected, the infarct size was markedly larger in the model group compared with the sham group, whereas silencing TNF expression led to an even greater infarct size, suggesting a protective role of TNF in mitigating myocardial injury (Figure 4B). Histopathological analyses revealed extensive structural alterations in post-MI hearts, further implicating TNF in myocardial remodeling (Figure 4C). Echocardiographic assessments demonstrated significant impairments in EF, CO, FS, and SV in the model group compared with sham controls, whereas RNAi-mediated TNF silencing resulted in further functional deterioration (Figure 4D). Serum biomarkers of myocardial injury, including CK, CK-MB, and LDH, were elevated in the model group and further increased upon TNF knockdown (Figure 4E). Likewise, HF-related biomarkers ANP, BNP, and Ang-2 exhibited group-dependent differences, highlighting TNF’s regulatory role (Figure 4F). Microvascular density was reduced in the model group relative to sham controls and further decreased in the RNAi-TNF group (Figure 4G). In the sham group, BrdU^+^/CD31^+^ regenerating ECs were scarce, whereas a marked increase in newly formed CMECs was observed in the model group, however, this increase was significantly attenuated following TNF silencing (Figure 4H). Western blot analysis confirmed elevated expression of TNF pathway proteins (TNF-α, c-MYC, and c-FOS) in the model group, with a pronounced decrease following RNAi-mediated TNF knockdown (Figure 4I). Taken together, these *in vivo* results provide strong evidence that TNF signaling plays a pivotal role in EC proliferation and angiogenesis in the context of HF after MI. Targeted modulation of TNF may represent a promising therapeutic strategy to facilitate myocardial repair and improve cardiac outcomes.

### 2.5 Molecular docking screening of traditional Chinese medicines potentially targeting TNF

**FIGURE 5:**
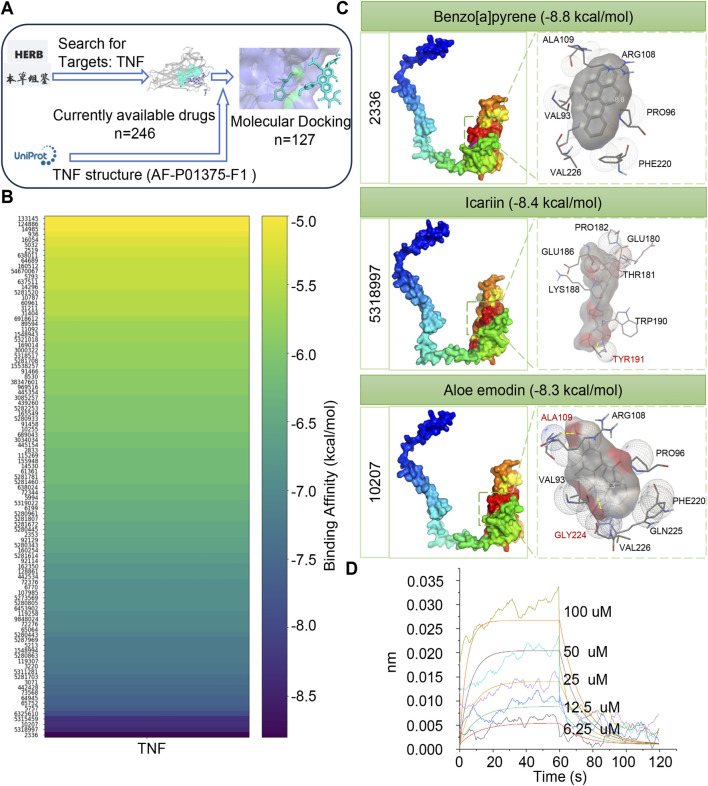
Molecular docking screening of traditional chinese medicines potentially targeting TNF. **(A)** Flowchart of the GEO database for online data retrieval. **(B)** Binding Affinity Heatmap of TNF with small molecules (binding affinity < −5.0). **(C)** Visualization of molecular docking of TNF with small molecules. **(D)** Biolayer Interferometry of icariin and TNF-a.

To identify TCM-derived compounds capable of promoting CMEC proliferation via the TNF signaling pathway, a large-scale molecular docking analysis was performed based on the HERB database. A total of 246 candidate compounds were initially retrieved from the database. Following rigorous computational filtering based on the availability of 3D structural coordinates and ligand preparation compatibility, 127 molecules were successfully prepared and docked with TNF (Figure 5A; Supplementary Figure S2). A binding-affinity heatmap was subsequently generated to visualize the interaction strength between TNF and the docked small molecules (Figure 5B). Compounds with binding energies below −5.0 kcal/mol, a widely accepted empirical threshold indicating stable non-covalent interactions, were highlighted, with a color gradient representing the relative binding affinity, more negative values indicated stronger binding interactions (Supplementary Figure S3). Representative docking conformations of TNF with selected molecules are shown in Figure 5C. Benzo[a]pyrene exhibited the strongest binding affinity (−8.8 kcal/mol), followed by ICA (−8.4 kcal/mol) and aloe-emodin (−8.3 kcal/mol). However, due to the well-documented carcinogenicity of benzo[a]pyrene, it is unsuitable for therapeutic application. To experimentally validate the reliability of these computational docking predictions, we examined the binding of ICA to TNF-a using Bio-Layer Interferometry (BLI), and the two combined with an affinity of 3.57E-05 (KD value), suggesting that ICA exerts its pharmacodynamic effects through direct action on TNF-a (Figure 5D). Consequently, ICA was prioritized as a promising candidate for promoting EC proliferation through TNF pathway modulation.

### 2.6 Icariin binding enhances the structural stability and conformational compactness of TNF

**FIGURE 6:**
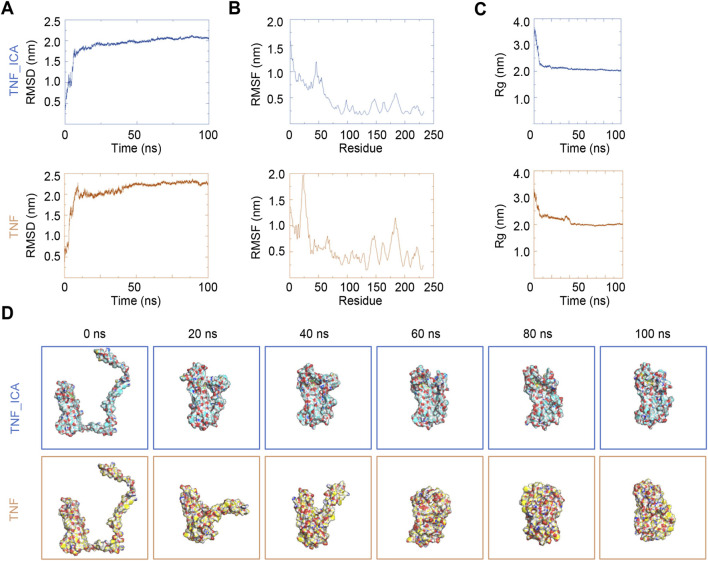
ICA promotes structural stabilization of TNF. **(A)** Root-mean-square deviation of TNF and ICA. **(B)** Root-mean-square fluctuation of TNF and ICA. **(C)** Radius of gyration of TNF and ICA. **(D)** Typical diagram of TNF and ICA during molecular dynamics simulations. ICA, Icariin.

In Figure 6A, the root-mean-square deviation (RMSD) of TNF in complex with ICA (TNF–ICA) and TNF alone is presented over a 100 ns molecular dynamics simulation (MDs). For TNF–ICA, the RMSD increased rapidly during the initial phase and subsequently stabilized at approximately 2.0 nm, indicating that the complex reached a stable conformational state following an initial equilibration period. By contrast, the RMSD of TNF alone stabilized at a slightly higher value (approximately 2.2–2.3 nm), suggesting that ICA binding contributes to enhanced structural stability of TNF. The root-mean-square fluctuation (RMSF) profiles of individual TNF residues are depicted in Figure 6B. Across most residues, the RMSF values of TNF–ICA were consistently lower than those of TNF alone, indicating reduced residue flexibility upon ICA binding. This reduced flexibility likely contributes to the overall stabilization of the TNF structure. The Rg of TNF–ICA stabilized at a lower value relative to TNF alone, which maintained a comparatively higher Rg. A lower Rg reflects a more compact protein conformation, further indicating that ICA binding promotes a more compact and stable TNF structure (Figure 6C). Representative structural conformations of TNF with and without ICA at multiple time points are provided in Figure 6D. Collectively, the RMSD, RMSF, and Rg analyses, together with visual structural assessments, consistently demonstrate that ICA binding enhances the structural stability and compactness of TNF. This stabilization may have functional implications for TNF-mediated promotion of EC proliferation.

### 2.7 Icariin promotes endothelial cell proliferation via TNF signaling pathway

**FIGURE 7:**
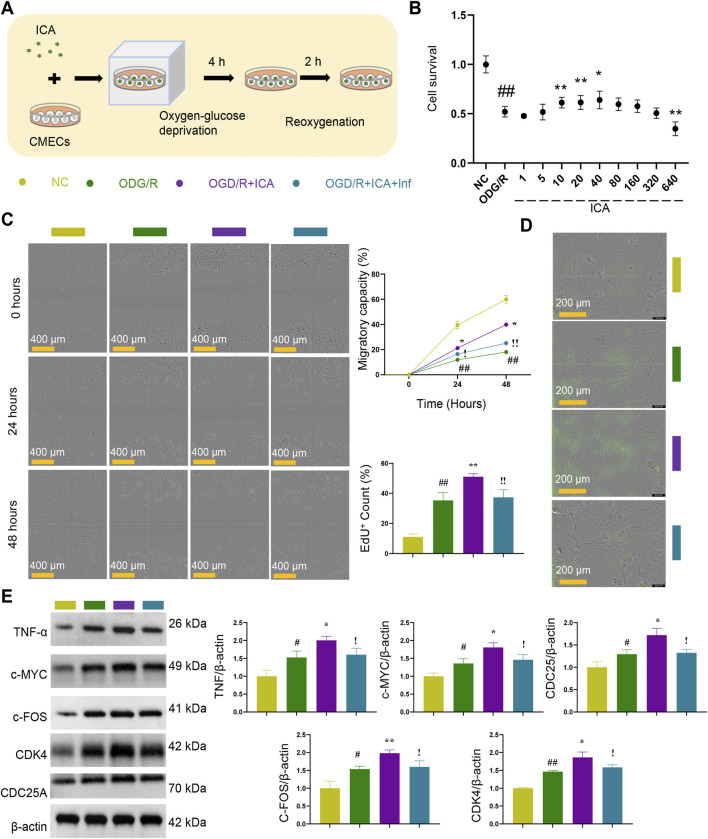
*In vitro* experiments confirm ICA promotes endothelial cell proliferation via TNF signaling pathway. **(A)** Schematic diagram of *in vitro* cell experiments of ICA. **(B)** Cell survival was induced with the addition of ICA (*n* = 6). **(C)** Migratory capacity was induced with the addition of ICA (*n* = 6). **(D)** EdU^+^ cells were induced with the addition of ICA (*n* = 3). **(E)** Western blot of TNF signaling pathway in each group (*n* = 3). Data are presented as the means ± SD. ICA, Icariin. Compared with NC group, ^#^ *p* < 0.05, ^##^ *p* < 0.01. Compared with OGD/R group, ^*^ *p* < 0.05, ^**^ *p* < 0.01. Compared with OGD/R + ICA group, ^!^ *p* < 0.05, ^!!^ *p* < 0.01.

To investigate whether ICA promotes CMECs survival via the TNF pathway, cells were treated with ICA under OGD/R conditions, with or without the addition of the TNF inhibitor infliximab (Figure 7A). Treatment with ICA significantly increased CMEC survival compared with OGD/R group (Figure 7B). ICA treatment enhanced migratory capacity over time, with the OGD/R + ICA group exhibiting a higher relative wound density at 24 and 48 h compared with OGD/R group. The addition of infliximab diminished the ICA-mediated improvement in migratory capacity (Figure 7C). The OGD/R + ICA group displayed a markedly higher percentage of EdU^+^ cells relative to OGD/R groups, whereas infliximab treatment reduced this proliferative effect (Figure 7D). The trend of Western Blot is consistent with that of Immunofluorescence (Figure 7E). Taken together, these *in vitro* findings strongly support that ICA promotes CMEC survival, migration, and proliferation, and that these beneficial effects are at least partially mediated via activation of the TNF signaling pathway. Inhibition of TNF with infliximab attenuates ICA-induced EC proliferation, underscoring the pathway’s mechanistic relevance.

## 3 Discussion

This study had provided comprehensive insights into the role of the TNF signaling pathway in EC proliferation, as well as the potential of ICA in modulating this process. The *in vitro* and *in vivo* experiments consistently demonstrated the crucial role of the TNF signaling pathway in the coordination of EC proliferation. In the *in vitro* setting, the addition of TNF inhibitors led to a significant reduction in cell survival, migratory capacity, and the expression of TNF pathway-related mRNA and proteins. This indicates that TNF is essential for maintaining the viability and regenerative capacity of ECs under OGD/R conditions. The *in-vivo* experiments further validated these findings. Rats with HF showed differences in infarct size, cardiac function, and biomarker levels, which were further exacerbated in the RNAi-TNF group, suggesting that modulating the TNF pathway can have a positive impact on the heart’s recovery process, likely through promoting EC proliferation.

The molecular-level investigations, such as GO and KEGG enrichment analyses, found the TNF signaling pathway as a key player in the complex network of biological processes related to HF and EC proliferation. Previous studies have unequivocally demonstrated the correlation between the TNF signaling pathway and the severity of HF, consistent with our bioinformatics findings. Furthermore, we observed that the upregulation of key genes in the TNF signaling pathway following HF may exhibit a significant association with angiogenesis. The enrichment of core genes in the TNF pathway and the demonstration of its regulatory effect on the cell cycle, provided mechanistic insights into how TNF promotes EC proliferation. Existing studies have developed a large number of drugs targeting TNF, including inhibitors and agonists, which can alleviate cardiac injury from aspects such as TNF transcription, translation, and function (He et al., 2005). A study has found that resveratrol oligomers with a 2,3-diaryllindanone scaffold can inhibit the production of TNF-α, which can be used for the treatment of ulcerative colitis, and relevant compounds have been optimized accordingly (Tang et al., 2022). Multi-omics research has revealed that TNF-α is a key driving factor for immune cell dysfunction in sepsis. Some research has also reviewed the development process of TNF inhibitors and elaborated on their practical applications as a successful paradigm of translational medicine (Bai et al., 2024). In addition, it has been discovered that the inhibitor of IKKε, SIKE, can exert an anti-nonalcoholic steatohepatitis (NASH) effect by blocking the activation of the MAPK signaling pathway, and there is an association between this process and the TNF signaling pathway (Wang et al., 2020; Arias et al., 2024). Moreover, a study from the perspective of immunometabolism has uncovered a new mechanism of TNF inhibitors in the treatment of Behcet’s disease, that is, TNF-α can induce the expression of the TRPM2 channel in neutrophils, while TNF inhibitors can block this effect, providing new targets for the treatment of related diseases (Perez-Ruiz et al., 2019; Oh et al., 2022; van der Houwen and van Laar, 2020; Tong et al., 2019). Importantly, as highlighted by recent advancements in the field, the recovery of the endothelial network following myocardial infarction is inextricably linked to specific immune mechanisms within the cardiac microenvironment. Immune cells, particularly macrophages and neutrophils, undergo dynamic phenotypic shifts that directly dictate the success of endothelial cell cycle reentry and subsequent vascular repair (Tombor et al., 2025). While our current findings emphasize the direct impact of ICA on CMECs through the structural stabilization of TNF-α, it is highly plausible that this stabilized conformation also beneficially modulates the broader immune landscape. By preventing excessive inflammatory cytotoxicity while preserving necessary pro-angiogenic immune cascades, ICA-mediated TNF stabilization may foster a highly permissive, pro-regenerative immune microenvironment. Future investigations should further dissect the crosstalk between ICA-stabilized endothelial networks and specific immune cell polarizations during cardiac tissue repair.

TCM has a long history in the treatment of cardiovascular diseases. However, whether TCM can affect cardiac function through TNF still remains to be explored. The process of screening TCM-related small molecules that target TNF through molecular docking. The binding-affinity heatmap and molecular-docking visualizations helped identify potential compounds, such as benzo[a]pyrene, TNF - ICA, and aloe emodin, with high affinity for TNF. This screening process is of great significance for exploring new therapeutic agents that can modulate the TNF pathway to promote EC proliferation. ICA emerged as a compound of particular interest. Parameters such as RMSD, RMSF, and Rg indicated that TNF in complex with ICA had a more stable and compact structure compared to TNF alone. This structural stabilization may be a crucial step in modulating the function of TNF. ICA’s therapeutic efficacy has been linked to multiple pathways in previous studies, including TGF-β1/Smad-mediated anti-fibrotic effects, antioxidant activity, and modulation of immune responses (Jia et al., 2023). This underscored ICA’s pleiotropic nature, which is consistent with the complexity of disease pathogenesis. TNF, a factor that plays a crucial role in the development of cardiovascular diseases, functions in the form of a trimer through TNFR1 and TNFR2 to conduct signaling, and its structure is closely related to its function (Hobson et al., 2023; Ou et al., 2023). Existing studies have identified small molecule inhibitors, such as the UCB series of compounds, that can stabilize the asymmetric conformation of the TNF trimer (O'Connell et al., 2019; McMillan et al., 2021). These compounds bind to the pocket in the core of the TNF trimer, distort the trimer structure, alter the receptor-binding sites, reduce the binding affinity of TNFR1 at one site, decrease the number of receptors bound to it, and thus inhibit TNF signaling. Crystal structures showed that the TNF trimer bound to the compound forms a specific complex with TNFR1(39, 40). Relevant studies also indicated that soluble TNF and TNFR1 can self - assemble into a network, and the compounds can inhibit the formation of this network, affecting TNF function. These studies have revealed the impact of TNF structural changes on its interaction with TNFR1 and its signaling function, providing an important basis for the development of small molecule drugs for the treatment of cardiovascular diseases (O'Connell et al., 2019; McMillan et al., 2021; Qaiser et al., 2021; Zhang et al., 2024).

The *in vitro* experiments further confirmed that ICA promotes EC proliferation via the TNF signaling pathway. Our *in vitro* results demonstrated the robust rescue effect of ICA at 40 μmol/L against OGD/R-induced injury. This concentration aligns with established pharmacological consensus, where doses up to 40–50 μmol/L have been extensively validated to be safe and devoid of baseline cytotoxicity in endothelial models, maximizing therapeutic efficacy without off-target toxicity (Hu et al., 2015). Infliximab is a TNF-neutralizing antibody that specifically binds to TNF-α, thereby blocking its interaction with receptors and inhibiting the activation of the TNF signaling pathway (Jan et al., 2025). ICA increased cell survival, migratory capacity, and the number of proliferating ECs (EdU^+^ cells). However, when the TNF pathway was inhibited by infliximab, the beneficial effects of ICA were reduced. This indicates that ICA exerts its promoting effect on EC proliferation by acting on the TNF signaling pathway. While BLI and Infliximab rescue experiments provide robust evidence that ICA binds to TNF-α and that the subsequent pro-proliferative effects are TNF-dependent, the precise intracellular signal transduction cascade remains incompletely mapped. The direct link between the ICA-stabilized TNF conformation and the immediate transcriptional activation of downstream targets like c-MYC and c-FOS is currently inferred from our KEGG and GEO pathway analyses. Further in-depth mechanistic studies, such as receptor-binding kinetics assays and direct promoter-binding experiments (e.g., ChIP-seq), are required to definitively map the intracellular molecular events triggered by this stabilized conformation.

A central question raised by our findings is how the structural stabilization of TNF-α by ICA translates into a “signaling switch” from its classical pro-inflammatory/apoptotic roles to a pro-regenerative outcome (Lightwood et al., 2021; Zhang et al., 2023). Theoretically, the biological effects of TNF-α are governed by its trimeric stability and the specific recruitment of its two distinct receptors, TNFR1 and TNFR2. While TNFR1 is ubiquitously expressed and often associated with inflammatory and apoptotic cascades, TNFR2 is primarily expressed in specific cell types, including ECs, and is known to mediate tissue repair, cell survival, and angiogenic signaling (Wang and Al-Lamki, 2013).

Our MD simulations demonstrated that ICA binding significantly enhances the conformational compactness and thermodynamic stability of the TNF-α trimer. In the context of the cardiac microenvironment post-MI, such structural stabilization may facilitate a biased signaling mechanism (Hattori et al., 2010). We hypothesize that the ICA-stabilized TNF-α trimer possesses a higher spatial affinity for TNFR2 or promotes the formation of stable TNFR2 signaling complexes, thereby preferentially activating the c-MYC/c-FOS/CDK4 cell-cycle axis. This hypothesis is supported by our observation that pharmacological inhibition of TNF binding by infliximab completely abolished the ICA-induced upregulation of cell-cycle proteins, confirming that the regenerative effect is strictly TNF-dependent. Although receptor-specific knockdown was not performed in this study, the robust induction of proliferation-related markers strongly suggests a shift toward TNFR2-mediated regenerative pathways. Future studies utilizing TNFR1/2-deficient models will be instrumental in further dissecting this conformational bias.

These findings have several important implications. From a clinical perspective, they have the potential to serve as therapeutic targets for the treatment of HF subsequent to myocardial infarction. By promoting EC proliferation, it may be possible to improve cardiac function and prognosis. From a research perspective, the identification of ICA and its mechanism of action opens up new avenues for the development of TCM-based therapies. Future studies could focus on optimizing the use of ICA, exploring its long-term effects, and further elucidating the detailed molecular mechanisms involved in the TNF-ICA-EC proliferation axis.

Several limitations should be acknowledged. While our *in vivo* experiments utilizing AAV9-mediated TNF silencing robustly demonstrated the physiological necessity of the TNF signaling pathway in post-MI microvascular repair, we did not directly evaluate the *in vivo* therapeutic efficacy of ICA administration in the MI rat model. Although our extensive *in vitro* and computational data strongly support ICA as a promising conformational stabilizer of TNF-α, the absence of direct *in vivo* drug treatment data limits the immediate translational impact of our findings. The complex pharmacokinetics, optimal systemic dosing, and potential off-target effects of ICA in a whole-body ischemic environment remain to be elucidated. It should be clarified that this study primarily focuses on the autocrine secretion of TNF by CMECs, excluding the influence of other cells, and mainly analyses the effect of TNF autocrine secretion on angiogenesis. Furthermore, we acknowledge that evaluations based solely on morphological assessments via H&E staining are insufficient to fully capture the complex, multifaceted immune responses driving tissue repair (Kaprin et al., 2022). The successful regeneration of the endothelial network requires orchestrated crosstalk with specific immune populations, including the dynamic polarization of macrophages, the early recruitment of neutrophils, and the regulatory functions of mast cells. Because our current morphological analyses could not properly assess these distinct immune subsets, a critical next step will be to incorporate comprehensive immunohistochemical and flow cytometric evaluations. Future studies must determine exactly how ICA-mediated TNF-α structural stabilization alters the infiltration, polarization, and functional states of these specific immune cells within the peri-infarct microenvironment. Besides, the *in vitro* experiments were conducted under controlled conditions. While the rat LADL model is a standard representation of ischemic heart failure, rodent cardiovascular physiology and immune responses do not perfectly mirror those of humans, limiting direct clinical generalizability. It is necessary to further validate the findings in more complex *in-vivo* models and human clinical trials. Additionally, while the molecular docking and simulation studies provided valuable insights into the interaction between ICA and TNF, more in-depth structural and functional studies are needed to fully understand the mechanism. Future research could also explore the combination of ICA with other compounds or therapies to enhance the therapeutic effect. Furthermore, while we utilized infliximab to confirm the necessity of the TNF pathway in ICA-mediated endothelial regeneration *in vitro*, we acknowledge the pharmacological complexity of this monoclonal antibody. Infliximab effectively neutralizes soluble TNF (sTNF) but also binds to membrane-bound TNF (tmTNF), which can potentially trigger complex reverse signaling cascades. Consequently, our current *in vitro* model cannot fully dissect the differential contributions of sTNF versus tmTNF to endothelial proliferation, nor does it perfectly capture the long-term spatiotemporal dynamics of TNF signaling in the remodeling heart. Future investigations employing cleavage-resistant tmTNF mutant models or highly specific sTNF/tmTNF selective inhibitors will be required to precisely delineate this complex signaling dimensions.

In conclusion, this study has provided a comprehensive understanding of the role of the TNF signaling pathway in EC proliferation and the potential of ICA as a therapeutic agent. The findings lay a solid foundation for further research and the development of new treatments for heart-related diseases.

## 4 Methods

### 4.1 Animals and cells

SPF grade male healthy adult SD rats (200–240 g, 7 weeks old) were obtained from SiPeiFu (Beijing) Biotechnology Co., Ltd. [Animal certificate number: SCXK (Beijing) 2019-0010]. 24 rats were given chow and water-adapted feeding for 7 days and were randomly divided in Sham group and left anterior descending coronary artery ligation (LADL) group (Model).

In order to confirm TNF signaling pathway promotes EC proliferation, 24 rats were randomly divided in Sham group, Model group and RNAi-TNF group. Rats received a single intravenous injection of RNAi-TNF. This 2-week lead time was designed to allow for optimal viral transduction and robust knockdown of the target protein before the ischemic insult occurred. BrdU was administered intraperitoneally at a concentration of 50 mg/kg. The ethics committee of Xiyuan hospital approved the study (No. 2021XLC037, 29 March 2021).

To minimize stress and preserve animal wellbeing, which can otherwise negatively impact experimental outcomes (Klabukov et al., 2023), non-invasive identification methods were prioritized. The rats were marked and identified utilizing non-toxic fur and skin staining with dyes. This method was regularly monitored and reapplied as necessary during routine handling to maintain clear identification while avoiding the handling-induced anxiety associated with physical marking techniques.

Sample sizes were determined based on our previous experiences with the LADL model and preliminary power analyses to ensure adequate statistical power for echocardiographic and histological endpoints. Animals were randomly assigned to experimental groups using a computer-generated random number sequence. To minimize bias, investigators performing echocardiographic assessments, histological scoring, and data analyses were blinded to the group allocation of the animals.

After the experiment and before tissue collection, all rats were euthanized via intraperitoneal injection of an overdose of sodium pentobarbital at a dose of 100 mg/kg body weight. Following administration, the rats’ status was continuously monitored, with respiratory arrest, absence of corneal reflex, and cardiac arrest used as the criteria for confirming death, and subsequent tissue collection was performed only after death was confirmed. This protocol complies with animal experiment ethical standards and minimizes the suffering of the rats. The handling of rats during the experimental process and the study was reported following the ARRIVE guidelines.

CMECs were purchased from Procell Life Science & Technology Co., Ltd. (Lot: CP-R135). The experiment was divided into the normal control group (NC), Oxygen-Glucose Deprivation/Reoxygenation group (OGD/R) and OGD/R + Infliximab (100 ng/mL) group (OGD/R + Inf). In order to confirm ICA promotes EC proliferation, the experiment was divided into NC, OGD/R, OGD/R + ICA (40 μmol/L) group (OGD/R + ICA), OGD/R + ICA + Infliximab (100 ng/mL) group (OGD/R + ICA + Inf).

### 4.2 Experimental instruments

ALC-V8 small animal ventilator was purchased from Shanghai Alcott Biotechnology Co., Ltd. (China). MPIAS-500 Multimedia Color Pathology Graphic Analysis System was purchased from Jingzheng Hengbocheng Technology Development Co., Ltd. (China). The microplate reader (BioTekSYNERGY4) was purchased from Berten Instrument Co., Ltd. (USA). Biological signal acquisition and processing system (BL-420F) was purchased from Sichuan Chengdu Taimeng Technology Co., Ltd. (China). Electrophoresis instrument (powerpac200) was purchased from Bio-Rad Laboratories, Inc. (USA). Hitachi Transmission Electron Microscope (H-7500) was purchased from Hitachi Limited (Japan). The Gel imaging system (Tanon1600) was purchased from Shanghai Tianneng Technology Co., Ltd. (China). IncuCyte S3 live-cell imaging and analysis system was purchased from Sartorius AG (Germany).

### 4.3 Reagents and antibodies

ICA was purchased from National Institutes for Food and Drug Control (Cat No. Q6QC-6DTQ, China). TNF-α (Cat No.17590-1-AP), c-MYC (Cat No.67447-1-lg), c-FOS (Cat No. 66590-1-lg) were purchased from Proteintech Group, Inc (China). Recombinant Anti-Cdk4 antibody (Cat No. ab199728) and β-actin ((Cat No. ab8226) were purchased from Abcam Limited (UK). CDC25A (Cat No. ab199728) was purchased from Thermo Fisher Scientific (American). Creatine kinase (CK) Activity Assay Kit (Cat No. S03024), lactate dehydrogenase (LDH) Activity Assay Kit (Cat No. 03034) and creatine kinase isoenzymes Activity Assay Kit (Cat No. S03023) were purchased from Rayto life and analytical sciences Co., Ltd. (China). TNF alpha (TNF-a) (Cat No. HY-P70426A) was purchased from MedChemExpress (USA).

### 4.4 Establishment of animal model

The establishment of LADL was based on previous literature and refined according to the pre-testing and previous experimental experience of the research group (Liu et al., 2024; Li et al., 2024). SD rats were anaesthetized with 1% sodium pentobarbital (50 mg/kg) by intraperitoneal anesthesia and connected to the small-animal ventilator for artificial respiration and the small-animal electrocardiograph. Anesthesia was complete when muscle tension, corneal reflexes and response to skin pinching disappeared in the rats. The chest was then opened and the pericardium exposed by breaking the 3-4 ribs. Once the heart was exposed, a suture was placed approximately 2 mm below the bifurcation of the left anterior descending coronary artery and the drug/physiological saline was immediately injected through the tail vein. After stabilization for 10 min, the silk thread was threaded with a small polyethylene tube, tightening the silk thread, causing myocardial ischemia by pushing the tube method. The judgment criteria for ischemia success were adopted by using the electrocardiogram QRS amplitude to increase, ST-segment elevation and T wave towering or inversion. Notably, the sham operation group only threading without ligation, and other operations were the same as the model group.

### 4.5 Establishment of cell model

The CMECs were inoculated in a 96-well plate or a clear sterile plastic petri dish with Lids then put in the cell incubator with a 37 °C, 5% CO_2_ for 24 h. After discarding the old medium and washing with PBS for 2-3 times, in the NC group, the old medium was replaced with DMEM complete medium, and put the 96-well plate or the clear sterile plastic petri dish with Lids back in the incubator for 6 h waiting for testing. Meanwhile, after washing the old medium in the OGD/R group and each administration group, the old medium was replaced with a sugar-free medium in the OGD/R group, and drug and sugar-free medium in each administration group.

In an airtight box, continuously flowing 950 mL/L N_2_+50 mL/L CO_2_ mixed gas for 3 min to fully exhaust the residual oxygen. OGD/R and each administration group were cultivated in the cell incubator for 4 h. After the end of 4 h, the airtight box was opened and the sterile glucose solution was added to make the glucose concentration in the culture medium of 1 g/L. Cell viability was detected by cell-counting-kit-8 after the 96-well plate or the clear sterile plastic petri dish with Lids was placed back in the incubator for 2 h.

### 4.6 HE staining of myocardial tissue

SD rats were anesthetized by intraperitoneal injection of sodium pentobarbital. Subsequent experiments were performed after the rats were put under deep anesthesia. The thickness of 4 mm cardiac tissue was cut from the heart cross-section below the ligation line and fixed with 4% formalin for 24 h. Subsequently, the thick 4 mm cardiac tissue was embedded in paraffin, and HE staining was used to observe the pathological changes in myocardial tissue.

### 4.7 Measurement of myocardial infarct size by TTC staining

After the end of experiment, the rats were anesthetized by intraperitoneal injection of sodium pentobarbital. At the same time, the heart was quickly taken out and blotted dry with filter paper, and then placed in a −20 °C refrigerator for quick freezing for about 10–15 min 5 uniform slices with a thickness of about 1 mm were cut from the heart, and then the slices were quickly placed in a 6-well plate containing TTC staining solution and incubated at 37 °C for 15–30 min in the dark. After the color development of the section is completed, the slices were placed in a special solution for some time in 10% formalin to increase the contrast. After that, the stained tissues were taken out and rinsed with normal saline to an off the excess staining solution on the surface of the tissue. ImageJ was used to measure and analyze the infarct size and total myocardial area (percentage of infarct area = total infarct area/total myocardial area × 100%).

### 4.8 Detection of serum myocardial enzymes

After the end of experiment, the rats were anesthetized by intraperitoneal injection of sodium pentobarbital. The blood was collected from the abdominal aorta, left standing at room temperature for 1 h, and centrifuged at 3,500 rpm for 10 min in order to separate the serum. After taking the serum sample and preparing reagents according to the instructions of CK, CK-MB, and LDH kits and mixing the reagents with animal serum samples in proportion, the contents of CK, CK-MB and LDH were calculated by a microplate reader.

### 4.9 Cell viability assay

After each treatment, cell counting kit-8 was used to measure the viability of CMECs according to the standard protocol of the manufacturer. The absorbance was determined at 450 nm using a spectrophotometer.

### 4.10 Wound healing experiments

CMECs in logarithmic growth phase were taken, trypsin digested into single cell suspension, and inoculated into 96-well culture plates. On the next day, CMECs were scratched with 96-well woundmaker tool (Sartorius Cat. No. 4563) and rinsed with PBS for 3 times to remove scratched cells. Cells were grouped and treated according to the “Establishment of Cell Model”. After taking pictures, the images were opened with scratch wound analysis software (Sartorius) and the mean value of the distance between cells was calculated. To quantify cell migration, three randomly selected, non-overlapping fields along the scratch line were photographed for each well across six independent biological replicates (*n* = 6). The cell-free wound area in each image was manually traced and measured using ImageJ software (National Institutes of Health, Bethesda, MD, USA). The migratory capacity rate was calculated using the following formula: [Wound Area at 0 h - Wound Area at endpoint/Wound Area at 0h] ×100%.

### 4.11 Real-time PCR detection

The total RNA of cells in the blank control group, model group, and drug - administration group was extracted using the TRIzol method. The primers were synthesized by Wuhan Saiweier Biotechnology Co., Ltd., and the primer sequences are shown in Supplementary Table S2. After the concentration was detected, reverse transcription reaction was carried out, followed by fluorescence quantification. The reaction conditions were as follows: pre-denaturation at 95 °C for 30 s, denaturation at 95 °C for 15 s, annealing at 60 °C for 30 s, and extension at 60 °C for 30 s, with 40 cycles in total. A melting curve was generated from 65 °C to 95 °C, and the fluorescence signal was collected every 0.5 °C temperature increase. Three replicate wells were set for each group in the experiment.

### 4.12 EdU immunofluorescence staining

CMECs in the logarithmic growth phase were taken and treated according to the “Animals and Cells and Establishment of Cell Model”. Six replicate wells were set for each group. After the OGD/R treatment was completed, EdU working solution was added according to the instruction manual and incubated for 2 h. After the EdU labeling was completed, the culture medium was removed, and 1 mL of 4% paraformaldehyde fixative was added for fixation at room temperature for 15 min. After removing the fixative, the cells were washed three times with the washing solution. Then, permeabilization solution was added for permeabilization for 10 min. After removing the permeabilization solution, the cells were washed three times with the washing solution. Click reaction solution was added and incubated in the dark for 30 min. Then, the cells were placed in the Incucyte® Live-Cell Imaging and Analysis System for photographing. The maximum excitation wavelength was 495 nm, and the maximum emission wavelength was 519 nm. For quantitative analysis, five randomly selected fields of view per well were captured across three independent biological replicates (*n* = 3). The number of EdU-positive nuclei (active DNA synthesis) and total Hoechst-stained nuclei were counted using the “Analyze Particles” function in ImageJ software. The cellular proliferation rate was expressed as the percentage of EdU-positive cells relative to the total number of cells per field.

### 4.13 Molecular dynamics simulation

To screen for potential TNF-targeting compounds from the 246 candidates identified in the HERB database, 3D structures (SDF format) were downloaded from the PubChem database. Ligand preparation was performed using AutoDockTools, which included the assignment of proper protonation states, calculation of Gasteiger charges, and definition of rotatable bonds. Compounds lacking defined 3D coordinates or failing the energy minimization preparation step were excluded, resulting in a final library of 127 molecules for docking. Molecular docking of TCM with the proteins was performed using AutoDock Vina (Eberhardt et al., 2021; Trott and Olson, 2010). A binding energy cutoff of ≤ −5.0 kcal/mol was applied as an empirical baseline to distinguish potential active hits from non-specific background interactions. The structure of TNF was modeled using AlphaFold3, and molecular dynamics simulations of the compound-target complex were carried out using GROMACS (Jumper et al., 2021; Varadi et al., 2024; Tan et al., 2022; Páll et al., 2020). The protein was simulated using the CHARMM36 force field, and the TIP3P water model was employed to fill the water molecules. To neutralize the system, chloride ions and sodium ions were randomly added to the simulation box. The system was first subjected to energy minimization using the steepest descent algorithm, followed by equilibration for 500 ps under the isothermal-isochoric (NVT) condition and 1,000 ps under the isothermal-isobaric (NPT) condition. During equilibration and production runs, the temperature was maintained at 300 K using a V-rescale thermostat, and the pressure was isotropically regulated at 1.0 bar using the Parrinello-Rahman barostat. Long-range electrostatic interactions were computed using the Particle Mesh Ewald (PME) method, with a 1.2 nm cutoff applied for both short-range electrostatic and van der Waals interactions. All covalent bonds involving hydrogen atoms were constrained using the LINCS algorithm. Finally, a continuous 100 ns MD production simulation was performed on the complex with a time step of 2 fs, and structural coordinates were saved every 10 ps for downstream analysis. Given the absence of a co-crystallized ligand in the AlphaFold-modeled structure, this extensive MD simulation, alongside subsequent *in vitro* BLI binding assays, served as the orthogonal validation methodology to verify the stability and reliability of the static docking poses. GROMACS, VMD, and LigPlot^+^ were used for structural analysis and visualization (Meng et al., 2023; Laskowski et al., 2011).

### 4.14 Western blot analysis

To obtain the protein of rat myocardial tissue, the rats were anesthetized by intraperitoneal injection of sodium pentobarbital. The ischemic and peripheral myocardial tissue were collected under the ligature and cut into pieces with surgical scissors. The tissue cells were lysed with RIPA lysis buffer (containing protease inhibitors and phosphatase inhibitors). In order to obtain the protein of CMECs, adding and mixing a certain amount of PBS to the cell pellet, after the cells were suspended in PBS, the cell suspension was transferred to a glass homogenization tube in the ice-water mixture. Manually homogenizing for 3 min, the broken cell suspension can be used for measurement. The protein was transferred to the PVDF membrane by determining the concentration by BCA protein detection kit. The membranes were incubated with the primary antibodies against TNF-α, c-MYC, c-FOS, CDK4, CDC25A and β-actin overnight at 4 °C. Then, the membranes were washed with TBST buffer and incubated with secondary antibody in TBST buffer at 37 °C for 1 h. Finally, the membranes were washed and visualized using Super ECL Western Blotting Substrate, and the bands were scanned and quantified using the ImageJ system.

### 4.15 Bioinformatics analysis

The dataset GSE184649 was retrieved from the Gene Expression Omnibus (GEO) database. Principal component analysis (PCA) was performed to assess the global differences in gene expression between groups. Differentially expressed genes (DEGs) were screened using differential expression analysis tools (DESeq2), and a volcano plot was generated. Gene Ontology (GO) functional enrichment analysis and Kyoto Encyclopedia of Genes and Genomes (KEGG) pathway enrichment analysis were conducted on the DEGs (Kanehisa et al., 2025; Kanehisa, 2019; Kanehisa and Goto, 2000). Furthermore, the protein - protein interaction (PPI) network of core genes in the TNF signaling pathway was constructed using the STRING database.

### 4.16 Statistical analysis

All data are expressed as mean ± standard error of the mean. Statistical software uses SPSS 20.0 statistical data. The Student's t-test was used for comparisons between two independent groups. For comparisons involving three or more groups with a single independent variable, a one-way analysis of variance (ANOVA) was utilized. For data sets evaluating the interaction of two independent variables, a two-way ANOVA was performed, followed by Tukey’s multiple comparisons post-hoc test. P values less than 0.05 were considered significant. GraphPad Prism 9.0 software was used for statistical data, and the level of significance is indicated in the legend.

### 4.17 Data availability

The data supporting the findings of this study are available from the corresponding author upon request. Whole transcriptome RNA Sequencing data presented in this study are publicly available under the accession number GSE135312 at the National Center for Biotechnology Information advances science and health.

## Data Availability

The original contributions presented in the study are included in the article/Supplementary Material, further inquiries can be directed to the corresponding authors.

## Glossary

MI: myocardial infarction
TNF: tumor necrosis factor
ECs: endothelial cells
IL-1: interleukin-1
IL-6: interleukin-6
ECM: extracellular matrix
TCM: Traditional Chinese medicine
GEO: Gene Expression Omnibus
ICA: icariin
EF: ejection fraction
FS: fractional shortening
CO: cardiac output
SV: stroke volume
CK: creatine kinase
CK-MB: creatine kinase-MB
LDH: lactate dehydrogenase
ANP: atrial natriuretic peptide
BNP: brain natriuretic peptide
Ang-2: angiopoietin-2
BrdU: bromodeoxyuridine
CD31: cluster of differentiation 31
CMECs: coronary microvascular endothelial cells
PCA: Principal component analysis
HF: heart failure
GO: Gene Ontology
KEGG: Kyoto Encyclopedia of Genes and Genomes
OGD/R: oxygen–glucose deprivation/reoxygenation
RMSD: root-mean-square deviation
AAV: adeno-associated virus
MDs: molecular dynamics simulation
RMSF: root-mean-square fluctuation
Rg: gyration
NASH: nonalcoholic steatohepatitis
