# Supplementary material for: Icariin-mediated structural stabilization of TNF-α triggers a pro-regenerative signaling switch for endothelial recovery after myocardial infarction
Source: Front Pharmacol. 2026 May 8;17:1817340. doi: 10.3389/fphar.2026.1817340 (PMC13194572; doi:10.3389/fphar.2026.1817340)
Supplement: Supplementary file 1 [file DataSheet1.docx]

**SUPPLEMENTARY MATERIAL**

This file includes:

Supplementary Fig.1-6

Supplementary Table 1-2

**Supplementary Fig.1**

**
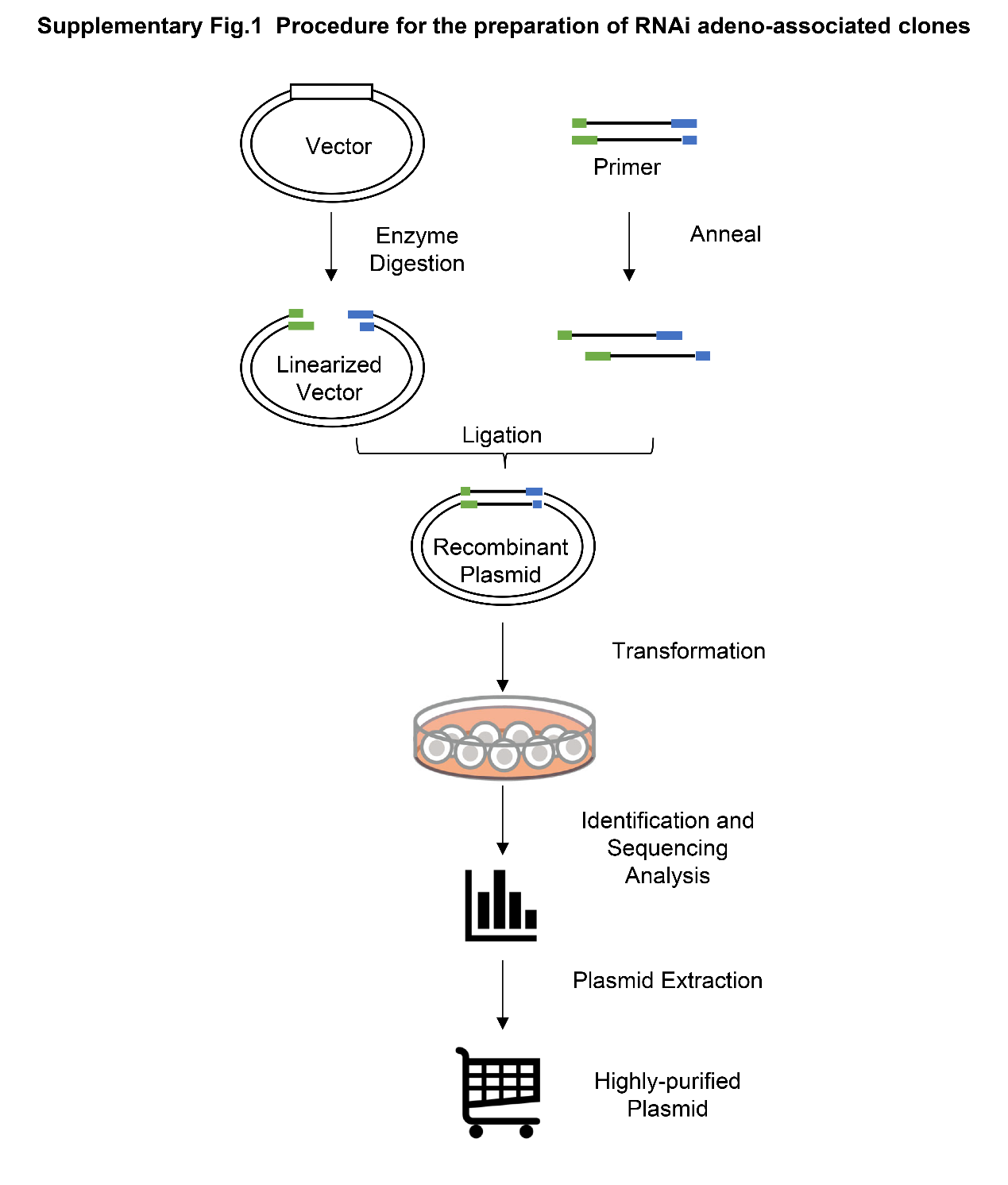
**

**Supplementary Fig.1 Procedure for the preparation of RNAi adeno-associated clones.**

**Supplementary Fig.2**


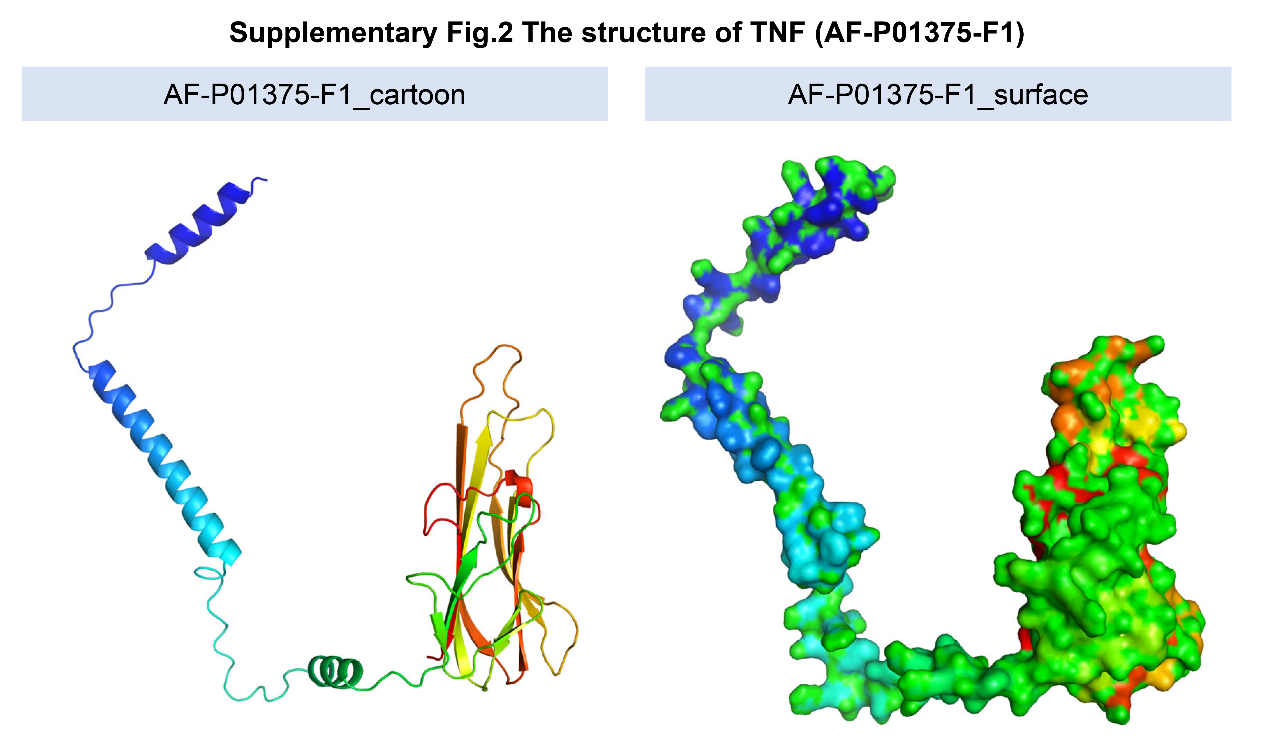


**Supplementary Fig.2 The structure of TNF (AF-P01375-F1)**

**Supplementary Fig.3**


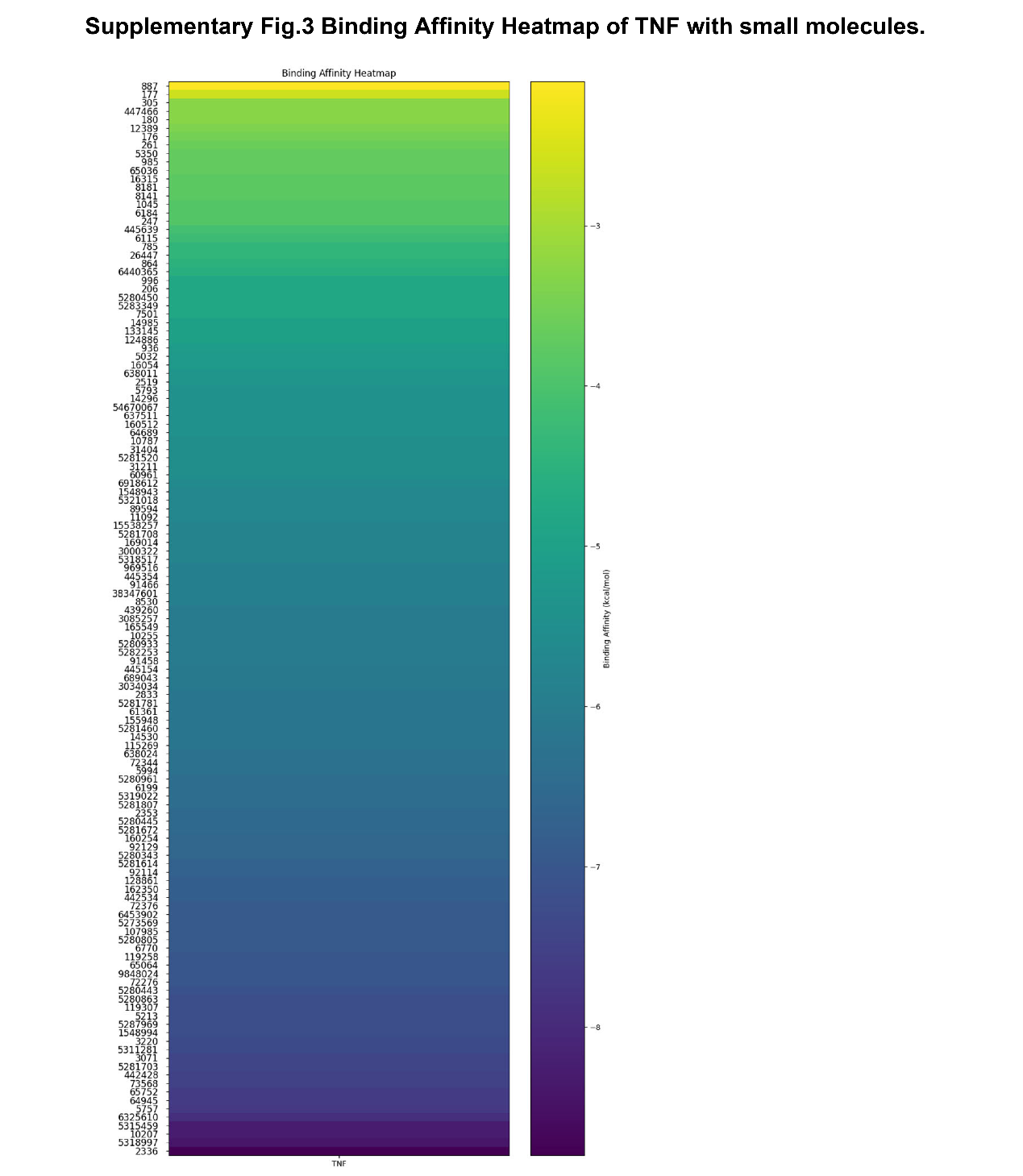


**Supplementary Fig.3 Binding Affinity Heatmap of TNF with small molecules.**

**Supplementary Fig.4**


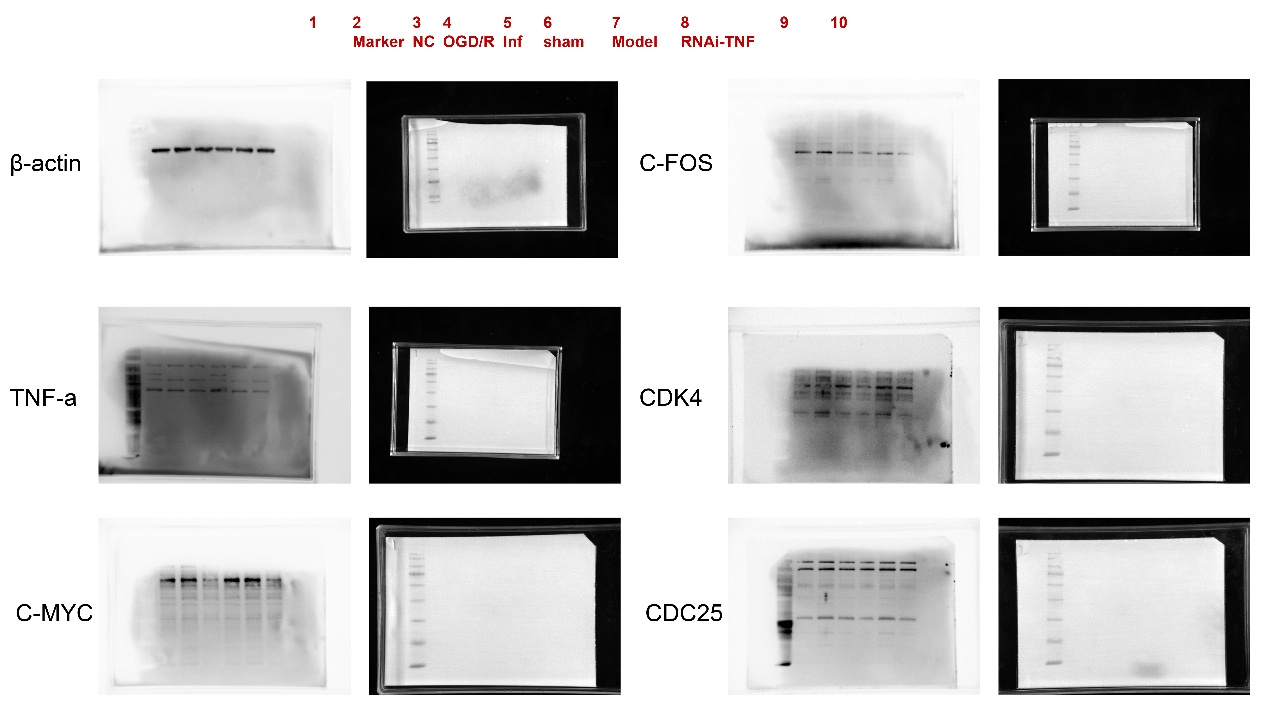


**Supplementary Fig.4 Western blot of *in vivo* and *in vitro* (sample 1).**

**Supplementary Fig.5**


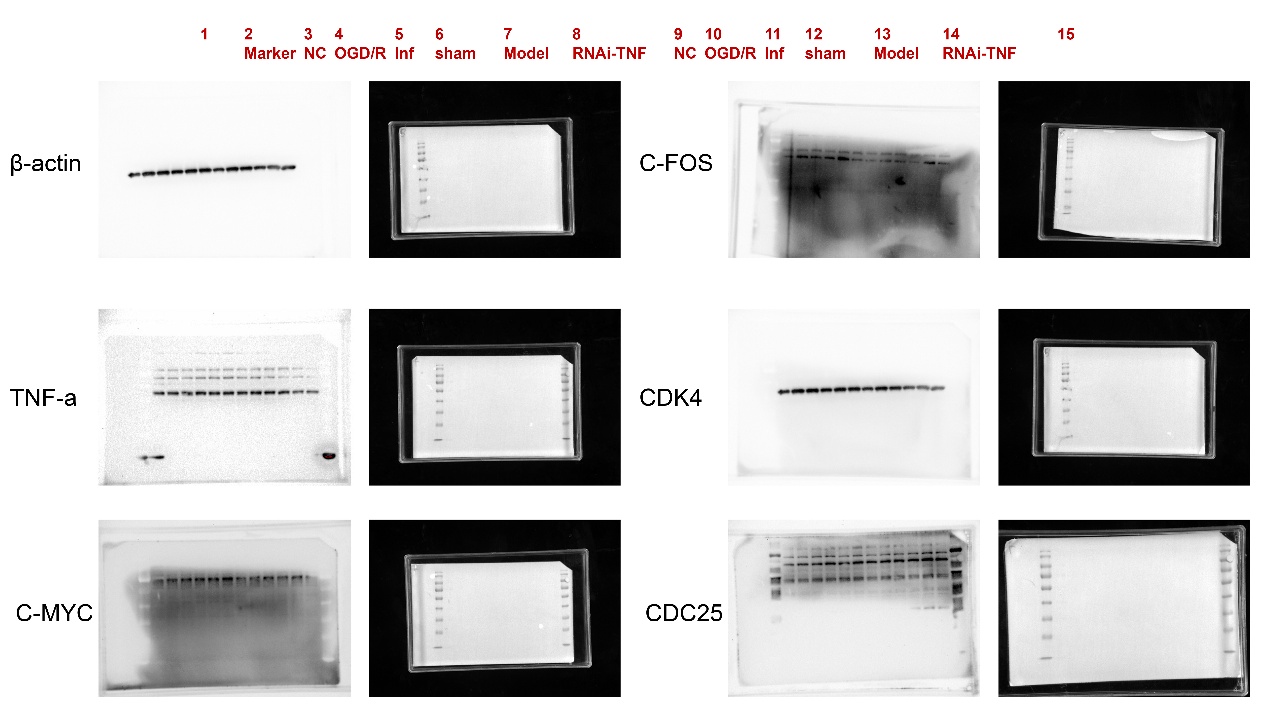


**Supplementary Fig.5 Western blot of *in vivo* and *in vitro* (sample 2 and 3).**

**Supplementary Fig.6**

**
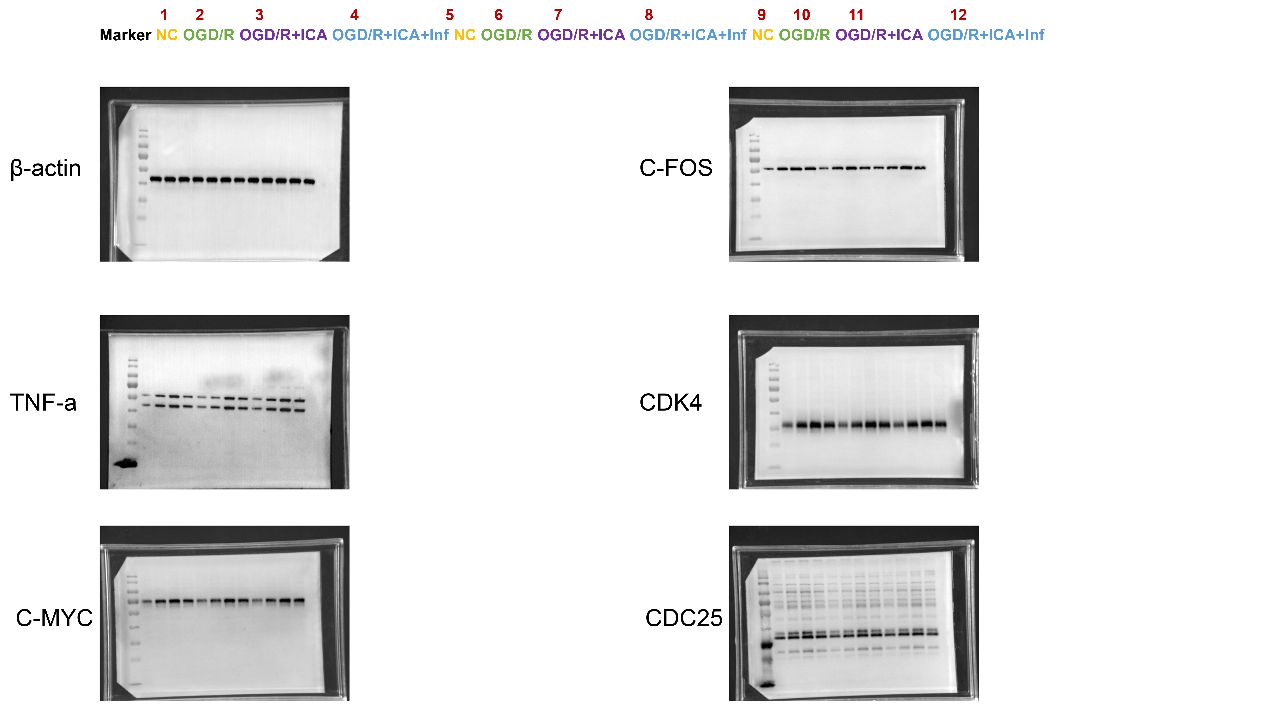
**

**Supplementary Fig.6 Western blot of normal control, OGD/R, OGD/R + ICA and OGD/R + ICA + Inf (sample 1 to 3).**

**Supplementary Table 1 Synthesis of oligo information**

| **NO.** | **5’** | **STEM** | **Loop** | **STEM** | **3’** |
| --- | --- | --- | --- | --- | --- |
| Tnf-RNAi(134689-2) | GACGAGCTGTACAAGGCTAGCTAACTGGAGGCTTGCTGAAGGCTGTATGCTG | TTGTCTTTGAGATCCATGCCA | GTTTTGGCCACTGACTGAC | TGGCATGGCTCAAAGACAA | CAGGACACAAGGCCTGTTACTAGCACTCACATGGAACAAATGGCCCCTCGAGAATCAACCTCTGGAT |

**Supplementary Table 2 Primer design for qPCR**

| primer name | 5’-3’ | length(bp) | ℃ |
| --- | --- | --- | --- |
| TNF-α | CCACCACGCTCTTCTGTCTACTG | 151 |  |
|  | TGGGCTACGGGCTTGTCACT |  |  |
| C-FOS | CTACAGTGACAGTTACGGACGAG | 163 | 60 |
|  | CAATGAAGAAAGAACGAGACCCA |  | 60 |
| C-MYC | TGGAGGAGACATGGTGAATCAG | 96 | 60 |
|  | AAGCCGCTCCACATACAGTCC |  | 60 |
| CDK4 | TATGTGGAGTGTTGGCTGTATCTTC | 320 | 60 |
|  | CCGGGTCACTTTCCTCCTTGT |  | 60 |
| CDC25A | GCAAGCGTGTCATTGTCGTG | 102 | 60 |
|  | GGTATTCATTGCCAAGCCTATCTC |  | 60 |
| GAPDH | CTGGAGAAACCTGCCAAGTATG | 138 | 60 |
|  | GGTGGAAGAATGGGAGTTGCT |  |  |
